# Economic and clinical burden from carbapenem-resistant bacterial infections and factors contributing: a retrospective study using electronic medical records in Japan

**DOI:** 10.1186/s12879-022-07548-3

**Published:** 2022-06-29

**Authors:** Shinobu Imai, Norihiko Inoue, Hideaki Nagai

**Affiliations:** 1 Department of Clinical Data Management and Research, Clinical Research Center, National Hospital Organization Headquarters, 2-5-21, Higashigaoka, Meguroku, Tokyo, 152-8621 Japan; 2 Department of Drug Safety and Risk Management, School of Pharmacy, Tokyo University of Pharmacy and Life Sciences, 1432-1, Horinouchi, Hachioji, Tokyo, 192-0392 Japan; 3 Department of Health Policy and Informatics, Tokyo Medical and Dental University Graduate School of Medicine, 1-5-45 Yushima, Bunkyo-ku, Tokyo, 113-8519 Japan; 4 Center for Pulmonary Diseases, National Hospital Organization Tokyo National Hospital, 3-1-1 Takeoka, Kiyose, Tokyo, 204-8585 Japan

**Keywords:** Carbapenem resistance, Carbapenem-susceptible infection, Length of hospital stay, Hospitalization cost, Electronic medical records

## Abstract

**Background:**

Antimicrobial resistance is a major threat to global health and the world economy. The economic burden of carbapenem-resistant infections has not previously been evaluated. We aimed to compare the potential economic burden and clinical outcomes between carbapenem-resistant infections and carbapenem-susceptible infections in Japan.

**Methods:**

We conducted a retrospective cohort study using electronic medical records. Patients aged 15 years or older and with the diagnosis of pneumonia, urinary tract infection, biliary infection, and sepsis were included. Multivariable regression models with random effects were used to estimate the impact of carbapenem resistance on cost, length of hospital stay, and in-hospital mortality.

**Results:**

Among the 9,517 patients, 86 (0.9%) had carbapenem-resistant (CR) infections. Compared to carbapenem-susceptible (CS) infections, the patients with the CR infections were significantly more likely to receive mechanical ventilation (37.2 vs. 21.2%, P-value = 0.003), antibiotics (88.4 vs. 63.0%, P-value < 0.001), and especially carbapenem (31.4 vs. 8.3%, P-value < 0.001), before the bacterial culture test positive. Significantly higher median costs were found for the CR infections than the CS infections in the categories of medications (3477 US dollars vs. 1609 US dollars), laboratory tests (2498 US dollars, vs. 1845 US dollars), and hospital stay (14,307 US dollars vs. 10,560 US dollars). In the multivariable regression analysis, the length of stay was 42.1% longer and the cost was 50.4% higher in the CR infections than in the CS infections. The risk of in-hospital mortality did not differ between the two groups (odds ratio 1.24, 95% CI 0.72–2.11), due to the small sample size. The result was robust with a similar trend in the analysis using the inverse probability treatment weighting method.

**Conclusions:**

Compared to carbapenem-susceptible infections, carbapenem-resistant infections were associated with a higher cost and a longer length of stay. Detailed cost analysis showed significant differences in the categories of medication, laboratory tests, and hospital stay. To our knowledge, this study is the first to assess the potential economic burden of carbapenem-resistant infections using a large hospital-based database.

**Supplementary Information:**

The online version contains supplementary material available at 10.1186/s12879-022-07548-3.

## Background

Antimicrobial resistance is a major threat to global health and the world economy [1]. Recent studies have shown that over 33,000 people die from infections caused by antibiotic-resistant bacteria in the European Union every year [2]. Carbapenem-resistant (CR) gram-negative bacteria, including carbapenem-resistant *Enterobacteriaceae*, are a matter of national and international concern because of their high levels of antimicrobial resistance and their association with high mortality [3]. The countries with the highest rates of CR infections between 2012 and 2015 included Greece, Italy, Romania, Croatia, Portugal, and Spain [1].

Previous studies have reported that CR infections not only increase the mortality risk but also cost more to treat than carbapenem-susceptible (CS) infections [4–6]. The additional length of hospital stay (LOS) has been estimated to range from 1.1 to 15.8 days, and the estimated additional total cost of hospitalization has been estimated to range from 1512 to 10,403 US dollars (USD) [4–6].

The Japan Nosocomial Infections Surveillance (JANIS), which is managed by the Japanese Ministry of Health, Labour, and Welfare (MHLW), is one of the largest surveillance systems in the world [7–9]. As of 2021, 2418 medical institutions voluntarily participate, providing monthly data on the occurrence of nosocomial infections, isolation of drug-resistant bacteria, and infections caused by drug-resistant bacteria. Tsuzuki et al. estimated that the number of deaths from bloodstream infection attributable to methicillin-resistant *Staphylococcus aureus* was 3.3 per 100,000 inhabitants, while that attributable to fluoroquinolone-resistant *Escherichia coli* was 3.1 per 100,000 inhabitants in 2017 [10]. These are the only published estimates of mortality due to antibiotic-resistant infections in Japan, and no estimate of mortality due to CR infection has been provided. Furthermore, the economic burden of CR infections has not previously been evaluated.

We aimed to compare the economic burden and clinical outcome of CR infections with those of CS infections in this multicenter observational study with electronic medical records (EMRs) in the National Hospital Organization (NHO) in Japan.

## Methods

### Study design and data source

We conducted a multicenter observational study using EMRs of 55 hospitals in the NHO database managed by the NHO Headquarters. The NHO has been established in April 2004 and is the largest hospital organization in Japan, including general acute care hospitals and specialized long-term care hospitals. The NHO has the administrative claims database (Medical Information Analysis databank; MIA) and the clinical information database (NHO Clinical Data Archives; NCDA) [11]. The MIA is the claims database based on the Diagnosis Procedure Combination/Per-Diem Payment System (DPC/PDPS) of case-mix patient classification and a lump-sum payment system for patients in Japan [12, 13]. The MIA contains patient information of age, sex, diagnosis, comorbidities, complications, medical procedures, medications, etc. based on the medical insurance system. The NCDA is based on the Standardized Structured Medical Record Information Exchange (SS-MIX), including medical charts, laboratory data, bacterial culture data, and many other data fields on daily basis [14, 15]. Additional details have been reported elsewhere [12, 13, 15, 16].

### Participants

Patients who had a hospitalization between April 2016 and March 2020 were included. Inclusion criteria were patients aged 15 years and older, and having a diagnosis of pneumonia, urinary tract infection (UTI), biliary infection, or sepsis with the administration of the intravenous antibiotics. The diagnoses were defined according to the International Classification of Diseases, 10th Revision (ICD-10) codes: pneumonia (A241, C349, J15[01569], J18[0–289], J690, J85[01], J958, J170), UTI (N1[0–2], N209, N390, T835), biliary and pancreatic infections (K8[0–7]), and sepsis (A241, A327, A415, A41[89], I301, I330, J209, J950, L029, M8699, T814). Bacterial culture tests positive from either specimen of urine, blood, or sputum after more than 3 days of hospitalization were defined as healthcare-associated infection. The first positive of the carbapenem-resistant pathogen from the bacterial culture test was considered the index positive result of the carbapenem-resistant (CR) bacteria. Patients who had tested positive for CR bacteria at least once were classified into the CR infection group. Patients whose culture tests were positive for only carbapenem-susceptible bacteria were classified as carbapenem-susceptible (CS) infection group. Carbapenem resistance was determined based on the results of antimicrobial susceptibility testing according to the JANIS definition [8].

**Fig. 1:**
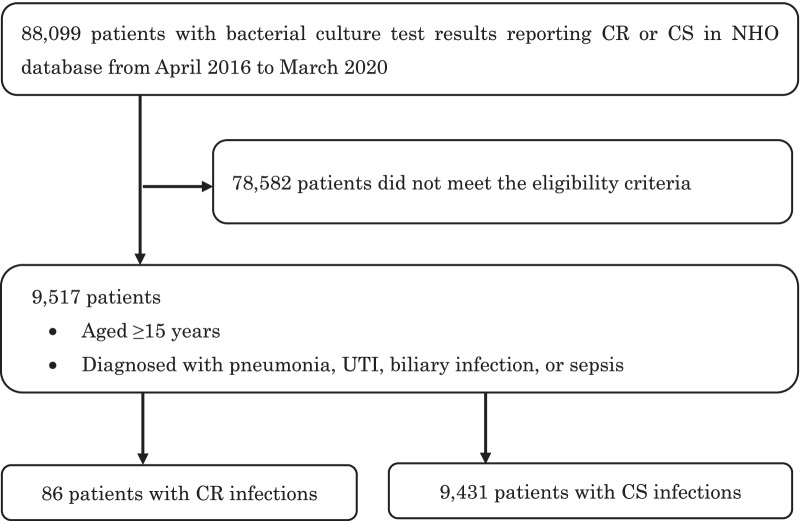
Patient selection flowchart. *CR* carbapenem-resistant, *CS* carbapenem-susceptible, *NHO* National Hospital Organization, *UTI* urinary tract infection

Some patients received special medical care with the public expenditure, such as incurable diseases, congenital diseases, and war victims, whose medical costs are fully covered by the government. In this study, none of the patients treated by the public expenditure met the inclusion criteria: in the NHO database, the number of publicly funded patients with carbapenem-susceptible or -resistant organisms detected by bacterial culture tests was 0.03% (305 of 88,099 in total at the first step of the patient inclusion flowchart in Fig. 1), and there were no relevant patients in our final analysis.

### Outcomes

We hypothesized that patients with CR infections would stay in the hospital longer and that their cost during hospitalization would be higher than that of patients with CS infections. The outcomes were total direct cost during hospitalization, in-hospital mortality, and total LOS.

### Variables

We extracted baseline of patient characteristics, medications, and medical procedures from the database: age, sex, body mass index (BMI), route of hospital admission, antibiotics, immunosuppressive drugs including chemotherapy and steroids, intensive care unit (ICU) admission, surgical procedure, dialysis, mechanical ventilation. In addition, we extracted the clinical data from the database related to bacterial infections: results of culture tests with organism type and antimicrobial susceptibility testing, white blood cell (WBC) count, body temperature (BT), and C-reactive protein (CRP) levels during hospitalization. The Charlson Comorbidity Index (CCI) scores were calculated according to the Quan’s coding algorithms [13], and used as a measure of the burden of chronic illness [17]. Imputation methods were used to estimate missing values in body weight, height, CRP level, WBC count, and BT. The following bacteria species were defined as research targets: *Pseudomonas aeruginosa, Acinetobacter spp., Escherichia coli, Klebsiella pneumoniae, Klebsiella oxytoca, Klebsiella aerogenes, Enterobacter cloacae, Citrobacter freundii, Proteus mirabilis,* and *Proteus vulgaris*. Following the JANIS guidelines [8, 10], the definition of resistance was based on the bacterial culture test. The carbapenem-resistance was defined by the result of antimicrobial susceptibility testing, as one of the above organisms with at least one determination of an ‘R’ (resistant) in the two algorithms (‘R’ result to imipenem-cilastatin sodium, or ‘R’ result to both meropenem hydrate and cefmetazole sodium). Hospital-level characteristics were grouped according to the number of beds: < 400, 400–500, and ≥ 500 beds.

### Statistical methods

To summarize patient characteristics, continuous variables were expressed as the mean and standard deviation (SD) or the median and interquartile range (IQR), depending on the distribution of variables. The Wilcoxon rank-sum test or the Welch test were used for assessing between-group differences. Categorical variables were expressed as proportions and compared using the chi-square test [18] To assess the impact of carbapenem resistance on in-hospital mortality, LOS, and cost, the hierarchical regression models were used with random effects accounting for the difference among hospitals [6, 19]. The covariates of interest were used for the adjustment in the models. The impact of carbapenem resistance on LOS and hospitalization cost was estimated as percent changes by linear regression with log-transformed values of these outcomes, and the impact on in-hospital mortality showed as odds ratios by logistic regression. The final regression model included age, sex, BMI, CCI, immunosuppressive drugs, antibiotic use before the bacterial culture test, ICU admission, undergoing a surgical procedure, disease type, and death, based on the methods in previous studies [4–6]. When death was the outcome of the model, it was not included as a covariate. The Akaike information criterion (AIC) was used for comparison among the unadjusted, adjusted, and multilevel models. To ensure the robustness of our results, we additionally adjusted for confounding by the inverse probability of treatment weighting (IPTW) with the method of the propensity scores overlap weights [20–25] (Additional file 1: Table S1–S3, Fig. S1).

The previous study indicated that the in-hospital mortality among patients with CS infection was 4.73% and with CR infection was 6.77% [5]. For this in-mortality rate, it was calculated that 2041 CR-infected and 2041 CS-infected patients had to be included in the analysis to obtain 80% statistical power to reject the null hypothesis that CS and CR in-hospital mortality rates are equal (Additional file 1: Appendix S1).

All analyses were performed using SAS version 9.4 statistical software (SAS Institute Inc., Cary, NC, USA) and R version 4.1.3 (R Foundation for Statistical Computing, Vienna, Austria. URL https://www.R-project.org/) [26].

## Results

A total of 9,517 eligible patients were included, of whom 86 (0.9%) had CR infections and 9,431 (99.1%) had CS infections (Fig. 1). Table 1 shows the types of organisms identified and their carbapenem susceptibility. Among the patients with CR infections, 22 (25.6%) had *Klebsiella aerogenes* and 18 (20.9%) had *Escherichia coli.*

**Table 1:** Bacterial species identified in culture tests and susceptibility to carbapenems

Organism, n (%)	Total (N = 9517)	CS infections (N = 9431)	CR infections (N = 86)
*Acinetobacter spp.*^†^	260 (2.7)	260 (2.8)	0 (0.0)
*Citrobacter freundii*	153 (1.6)	153 (1.6)	0 (0.0)
*Enterobacter cloacae*	794 (8.3)	778 (8.2)	16 (18.6)
*Escherichia coli*	3213 (33.8)	3195 (33.9)	18 (20.9)
*Klebsiella aerogenes*	430 (4.5)	408 (4.3)	22 (25.6)
*Klebsiella oxytoca*	608 (6.4)	604 (6.4)	4 (4.7)
*Klebsiella pneumoniae*	1913 (20.1)	1897 (20.1)	16 (18.6)
*Proteus mirabilis*	176 (1.9)	174 (1.8)	2 (2.3)
*Proteus vulgaris*	64 (0.7)	64 (0.7)	0 (0.0)
*Pseudomonas aeruginosa*	1906 (20.0)	1898 (20.1)	8 (9.3)

^†^*Acinetobacter baumannii*, *lwoffii* and others were included in *Acinetobacter* spp.

*CR* carbapenem-resistant, *CS* carbapenem-susceptible

Table 2 shows patients’ characteristics according to their carbapenem susceptibility status. Those with CR and CS infections were similar in age (74.9 ± 12.7 vs. 76.4 ± 12.5 years, P-value = 0.259), but patients with CR infections were significantly more likely to be men than those with CS infections (68.6 vs. 56.6%, P-value = 0.025). Among all patients, 4,587 were diagnosed with pneumonia, 1566 with sepsis, 3,278 with UTIs, and 1,323 with biliary infection. The prevalence of CR infections was 0.73% in patients with UTIs, 0.89% in patients with sepsis, 1.00% in patients with pneumonia, and 1.06% in patients with biliary infection. Compared to patients with CS infections, patients with CR infections were significantly more likely to receive mechanical ventilation (37.2 vs. 21.2%, P-value < 0.001) and antibiotics (88.4 vs. 63.0%, P-value < 0.001), including carbapenem (31.4 vs. 8.3%, P-value < 0.001), before the culture test.

**Table 2:** Patient characteristics according to carbapenem susceptibility of the infection

Variable	CS infections (N = 9,431)	CR infections (N = 86)	P-value
Age (years), mean ± SD	76.4 ± 12.5	74.9 ± 12.7	0.259
Age (years), n (%)			0.480
15–64	1331 (14.1)	16 (18.6)	
65–74	2098 (22.2)	19 (22.1)	
≥ 75	6002 (63.6)	51 (59.3)	
Sex, n (%)			0.025
Male	5334 (56.6)	59 (68.6)	
Female	4097 (43.4)	27 (31.4)	
BMI (kg/m^2^), mean ± SD	21.2 ± 4.2	20.8 ± 4.5	0.127
BMI (kg/m^2^), n (%)			
< 18.5	2517 (26.7)	28 (32.6)	0.221
18.5–25	5401 (57.3)	45 (52.3)	0.356
≥ 25	1513 (16.0)	13 (15.1)	0.816
WBC, mean ± SD	11,860.8 ± 7324.4	11,191.0 ± 6277.7	0.341
CRP, mean ± SD	12.2 ± 9.0	11.0 ± 8.8	0.149
BT, mean ± SD	38.5 ± 0.9	38.3 ± 1.0	0.007
Main diagnosis, n (%)			
Pneumonia	4541 (48.1)	46 (53.5)	0.324
Sepsis	1552 (16.5)	14 (16.3)	0.965
UTI	3254 (34.5)	24 (27.9)	0.200
Biliary infection	1309 (13.9)	14 (16.3)	0.522
Reason for hospitalization, n (%)			
Diseases of the circulatory system	2822 (29.9)	22 (25.6)	0.449
Neoplasms	2214 (23.5)	14 (16.3)	0.150
Diseases of the digestive system	1067 (11.3)	19 (22.1)	0.003
Charlson Comorbidity Index, n (%)			0.230
0 point	1041 (11.0)	13 (15.1)	
≥ 1 point	8390 (89.0)	73 (84.9)	
Comorbidity, n (%)			
Myocardial infarction	398 (4.2)	2 (2.3)	0.383
Cerebrovascular disease	2783 (29.5)	26 (30.2)	0.884
Congestive heart failure	1774 (18.8)	17 (19.8)	0.821
Rheumatic disease	274 (2.9)	6 (7.0)	0.026
Dementia	1353 (14.3)	7 (8.1)	0.102
Diabetes without complications	1972 (20.9)	25 (29.1)	0.064
Diabetes with complications	379 (4.0)	6 (7.0)	0.166
Mild liver disease	565 (6.0)	5 (5.8)	0.945
Moderate or severe liver disease	122 (1.3)	2 (2.3)	0.401
Peptic ulcer disease	951 (10.1)	4 (4.7)	0.095
Peripheral vascular disease	464 (4.9)	3 (3.5)	0.541
Chronic pulmonary disease	665 (7.1)	6 (7.0)	0.979
Paraplegia and hemiplegia	233 (2.5)	1 (1.2)	0.436
Renal disease	748 (7.9)	4 (4.7)	0.262
Cancer	3518 (37.3)	27 (31.4)	0.259
Metastatic carcinoma	1032 (10.9)	6 (7.0)	0.240
HIV	10 (0.1)	0 (0.0)	0.763
Healthcare utilization, n (%)			
ICU admission	2359 (25.0)	19 (22.1)	0.534
Surgical procedure	4634 (49.1)	48 (55.8)	0.218
Dialysis	512 (5.4)	5 (5.8)	0.875
Mechanical ventilation	1997 (21.2)	32 (37.2)	< 0.001
Immunosuppressive drug	3223 (34.2)	30 (34.9)	0.890
Antibiotics before the culture test	5937 (63.0)	76 (88.4)	< 0.001
Carbapenem before culture test	779 (8.3)	27 (31.4)	< 0.001
Number of beds, n (%)			0.021
< 400	2275 (24.1)	11 (12.8)	
400–500	3569 (37.8)	32 (37.2)	
≥ 500	3587 (38.0)	43 (50.0)	
Rout of admission, n (%)			0.070
From other wards	5 (0.1)	0 (0.0)	
From home	7,469 (79.2)	68 (79.1)	
From other hospital	1,175 (12.5)	13 (15.1)	
From long-term institutions	738 (7.8)	3 (3.5)	
Others	44 (0.5)	2 (2.3)	

*BMI* body mass index, *CCI* Charlson Comorbidity Index, *CR* carbapenem-resistant, *CRP* C-reactive protein, *CS* carbapenem-susceptible, *ICU* intensive care unit, *SD* standard deviation, *WBC* white blood cell, *BT* body temperature; *UTI* urinary tract infection

Table 3 shows the unadjusted differences in the outcomes between patients with CR and CS infections. Patients with CR infections had a longer LOS before the culture test (median 23.5 days, Interquartile range [IQR] 10.0–57.0 days vs. median 13.0 days, IQR 6.0–25.0) and a higher total hospitalization cost (median 20,077 USD, IQR 12,670–33,345 vs. median 27,501 USD, IQR 17,186–48,980) than did patients with CS infections. Significantly higher median costs were found in the CR infections for medications (median 3477 USD, IQR 1585–9716), laboratory tests (median 2498 USD, IQR 1666–4640), and hospital stay (median 14,307 USD, IQR 9790–22,471).

**Table 3:** Comparison of the economic and clinical outcomes according to carbapenem susceptibility of infecting organism

Variables	CS infections (N = 9431)	CR infections (N = 86)	P-value
Death, n (%)	2062 (21.9)	22 (25.6)	0.407
Length of hospital stay (days), median (IQR)			
Total	46 (29–74)	64 (39–99)	< 0.001
Before culture test	13 (6–25)	24 (10–57)	< 0.001
After culture test	27 (15–50)	30 (16–67)	0.132
Cost (USD), median (IQR)			
Total	20,077 (12,670–33,345)	27,501 (17,186–48,980)	< 0.001
Inter-consultations	143 (80–244)	163 (96–312)	0.238
Medications	1609 (736–3,571)	3477 (1585–9716)	< 0.001
Surgical procedures	2327 (362–9,377)	3761 (572–12,239)	0.196
Laboratory tests	1845 (1185–2930)	2498 (1666–4640)	0.002
Hospital stay	10,560 (6789–16,304)	14,307 (9790–22,471)	< 0.001

*CR* carbapenem-resistant, *CS* carbapenem-susceptible; *IQR* interquartile range, *USD* U.S. dollar (1 USD = 110 JPY, 2021); *SD* standard deviation

Table 4 shows the estimates of the hierarchical regression models for in-hospital death, LOS, and hospitalization cost of CR infections relative to CS infections. Compared to patients with CS infections, those with CR infections had a significantly longer LOS (42%, 95% confidence interval [CI] 29.1–55.2%, P-value < 0.001), and significantly higher hospitalization cost (50.4%, 95% CI 40.9–60.0%, P-value < 0.001). However, mortality was not significantly associated with carbapenem susceptibility status (odds ratio [OR] 1.24, 95% CI 0.72–2.11, P-value = 0.441). Older age, lower BMI, pneumonia, sepsis, and UTI were associated with an increased risk of death. Patients with pneumonia or sepsis had higher hospitalization costs than patients with other conditions.

**Table 4:** Impact of carbapenem resistance estimated by generalized linear models with random effect

Variables	Logistic regression^†^		Log-linear regression^†‡^			
	Death		LOS		Cost	
	OR (95% CI)	P-value	Percentage change (95% CI)	P-value	Percentage change (95% CI)	P-value
CR infections (Reference: CS infections)	1.24 (0.72–2.11)	0.441	42.1% (29.1–55.2%%)	< 0.001	50.4% (40.9–60.0%)	< 0.001
Age (years) (Reference: 15–64)						
65–74	1.40 (1.15–1.70)	< 0.001	–13.7% (− 18.8––8.6%)	< 0.001	–7.5% (− 11.9––3.2%)	< 0.001
≥ 75	1.80 (1.51–2.15)	< 0.001	–32.1% (− 36.9––27.3%)	< 0.001	–23.5% (− 27.6––19.4%)	< 0.001
Male (Reference: Female)	1.13 (1.01–1.26)	0.033	0.7% (− 3.1–4.4%)	0.728	–1.6% (− 4.7–1.6%)	0.334
BMI (kg/m.^2^) (Reference: < 18.5)						
18.5–25	0.66 (0.59–0.75)	< 0.001	–0.2% (− 4.5–4.0%)	0.911	9.0% (5.2–12.9%)	< 0.001
≥ 25	0.55 (0.46–0.65)	< 0.001	–1.4% (− 0.7–4.2%)	0.633	15.2% (10.5–19.9%)	< 0.001
Immunosuppressive drug	2.04 (1.82–2.28)	< 0.001	15.7% (11.9–19.4%)	< 0.001	27.8% (24.6–30.9%)	< 0.001
ICU	1.10 (0.97–1.26)	0.151	17.7% (13.2–22.2%)	< 0.001	45.3% (41.8–48.8%)	< 0.001
Surgical procedure	0.59 (0.52–0.66)	< 0.001	–14.4% (− 18.7––10.1%)	< 0.001	13.9% (10.2–17.6%)	< 0.001
Pneumonia	2.15 (1.83–2.54)	< 0.001	29.9% (24.7–35.0%)	< 0.001	17.8% (13.2–22.4%)	< 0.001
Sepsis	3.42 (2.90–4.04)	< 0.001	14.5% (9.0–20.1%)	< 0.001	18.1% (13.7–22.5%)	< 0.001
UTI	0.61 (0.52–0.73)	< 0.001	23.3% (18.0–28.7%)	< 0.001	2.9% (− 2.0–7.7%)	0.253
Biliary infection	0.87 (0.70–1.07)	0.178	18.4% (11.7–25.2%)	< 0.001	–0.1% (− 5.7–5.6%)	0.994
Death	–	–	6.3% (1.9–10.8%)	0.005	13.5% (10.0–17.0%)	< 0.001
Charlson Comorbidity Index ≥ 1	1.50 (1.01–1.50)	< 0.001	4.4% (− 0.7–9.6%)	0.091	7.5% (2.5–12.6%)	0.003
Antibiotics before culture test	1.05 (0.93–1.18)	0.474	27.7% (23.1–32.3%)	< 0.001	32.2% (27.9–36.5%)	< 0.001

^†^ The impacts of carbapenem resistance infections were calculated against carbapenem susceptible infections as reference

^‡^ Continuous outcomes of the LOS and cost were log-transformed. The estimated coefficient β of the carbapenem resistance was transformed as percent change per one-unit increase

^a^ Adjusted for age, sex, BMI, CCI, immunosuppressive drugs, antibiotic use before culture test, ICU admission, operation, disease type, and death (if outcome was death, it was excluded from the model)

^b^ Hospitals were treated as a random effect

BMI, body mass index; CCI, Charlson Comorbidity Index; CI, confidence interval; CR, carbapenem-resistant; CS, carbapenem-susceptible; ICU, intensive care unit; LOS, length of hospital stay; OR, odds ratio; UTI, urinary tract infection

Table 5 shows the unadjusted and adjusted differences in mortality, LOS, and hospitalization costs in patients with CR infections compared to patients with CS infections. In-hospital mortality did not differ significantly according to the carbapenem susceptibility status in the unadjusted and the multilevel regression (OR 1.23, 95% CI 0.76–2.00, P-value = 0.407; OR 1.24, 95% CI 0.72–2.11, P-value = 0.441, respectively). Relative to patients with CS infections, the LOS in patients with CR infections was estimated to be increased by 38.5% (95% CI 23.0–53.9%, P-value < 0.001), 25.4% (10.0–40.8%, P-value = 0.001) and 42.1% (29.1–55.2%, P-value < 0.001) using the unadjusted, adjusted, and multilevel models, respectively. Relative to patients with CS infections, the hospitalization cost of patients with CR infections was estimated to be increased by 50.1% (95% CI 37.8–62.5%, P-value < 0.001), 44.7% (33.3–56.2%, P-value < 0.001) and 50.4% (40.9–60.0%, P-value < 0.001) using the unadjusted, adjusted, and multilevel models, respectively.

**Table 5:** Comparison among unadjusted, adjusted and multilevel regression models for estimating the impact of carbapenem resistance

	Logistic regression^†^		
	OR (95% CI)	P-value	AIC
Death			
Unadjusted	1.23 (0.76–2.00)	0.407	10,007
Adjusted^a^	1.18 (0.70–1.99)	0.543	10,007
Multilevel^a,b^	1.24 (0.72–2.11)	0.441	8827

	Log-linear regression^†‡^		
	Percentage change (95% CI)	P-value	AIC
Total LOS			
Unadjusted	38.5% (23.0–53.9%)	< 0.001	106,802
Adjusted^a^	25.4% (10.0–40.8%)	< 0.001	106,214
Multilevel^a,b^	42.1% (29.1–55.2%)	< 0.001	104,230
Total Cost			
Unadjusted	50.1% (37.8–62.5%)	< 0.001	266,426
Adjusted^a^	44.7% (33.3–56.2%)	< 0.001	264,132
Multilevel^a,b^	50.4% (40.9–60.0%)	< 0.001	263,400

^†^The impacts of carbapenem resistance infections were calculated against carbapenem susceptible infections as reference

^‡^Continuous outcomes of the LOS and cost were log-transformed. The estimated coefficient β of the carbapenem resistance was transformed as percent change per one-unit increase

^a^Adjusted for age, sex, BMI, CCI, immunosuppressive drugs, antibiotic use before culture test, ICU admission, operation, disease type, and death (if outcome was death, it was excluded from the model)

^b^Hospital was treated as a random effect

*AIC* Akaike information criterion, *BMI* body mass index, *CCI* Charlson Comorbidity Index, *CI* confidence interval, *CR* carbapenem-resistant; *CS* carbapenem-susceptible, *LOS* length of stay, *OR* odds ratio

A propensity score analysis with inverse probability of treatment weighting was performed to check the influence of other variables, reduce the bias of between-group differences, and increase the robustness of the results. Even after balancing the covariates between CS and CR groups, the results were similar to the crude analysis: the risk of in-hospital mortality was not significant (OR 1.06, 95% CI 0.50–2.24, P-value = 0.880), and there was a significant percentage increase in the CR group for total costs (28.5%, 95% CI 23.6–33.4%, P-value < 0.001) and length of hospital stay (29.6%, 95% CI 24.6–34.6%, P-value < 0.001) (Additional file 1: Table S3).

## Discussion

This study revealed that the economic burden of CR infections was higher than that of CS infections. To our knowledge, the study is the first one to assess the potential economic burden of CR infections using a large hospital database. The CR infections were associated with a significantly longer total LOS and higher total hospitalization cost than CS infections in our study, even after the propensity score adjustment. On the contrary, no statistical difference was observed in mortality between CR and CS infections due to the small sample size in the CR infections as confirmed by the sample size calculation.

Detailed cost data and the analysis by multivariable regression and the inverse probability of treatment weighting adjustment showed that hospitalization charges, medications, and laboratory costs accounted for a large portion of the medical cost burden, which was also thought to be interrelated with the length of hospital stay before a culture test. The healthcare utilization of patients with CR infections was higher than that of patients with CS infections. The factors significantly associated with the total cost and longer total LOS: pneumonia, UTI, and the use of antibiotics, especially carbapenem, before the culture test. Other factors significantly associated with a higher hospitalization cost included ICU admission and the use of medication including antibiotics before the culture test. The findings are similar to the result from Rodriguez-Acevedo et al. that even asymptomatic patients colonized with the CR organisms had a six-fold higher mean hospitalization cost and  12 days longer mean LOS than patients  not colonized [27].

The LOS before the bacterial culture tests was significantly longer in patients with CR infection. However, the LOS after the bacterial culture tests did not differ on carbapenem susceptibility status in our study. The result suggests that the long total LOS in patients with CR infection is due to the long LOS before the bacterial culture tests are done. Additionally, the prevalence of antibiotic use, including carbapenem use, was higher among patients with CR infections than CS infections. Inappropriate use of antibiotics may increase the chance for carbapenem-resistant bacteria to emerge, which in turn will be associated with increased CR infections. A previous review by Righi et. al. reported that a longer hospital stay and previous exposure and/or a longer duration of exposure to carbapenems were more frequently associated with carbapenem resistance [28]. Patients with longer hospital stays are more likely to experience inappropriate use of antimicrobials. The findings suggest that appropriate use of antimicrobial agents and appropriate short completion of medical procedures are important to reduce the consequent emergence of carbapenem-resistant organisms. In general, prolonged patient hospitalization itself is also directly related to increased hospitalization costs. Appropriate medical treatment in the shortest possible time to control the outbreak of carbapenem-resistant bacteria is indeed reasonable in terms of both cost containment and length of hospital stay.

On the contrary, the association between CR infection and the in-hospital mortality rate was unclear in this study. Older age, lower BMI, use of immunosuppressive drugs, and more severe diseases such as pneumonia and sepsis were significant risk factors for in-hospital mortality, irrespective of the carbapenem susceptibility status of the infection. Previous reports showed that the 28-day in-hospital all-cause mortality rate of patients with Verona integron-encoded metallo-β-lactamase (VIM-positive) Pseudomonas aeruginosa infection was 22% higher than that of VIM-negative patients [29]. In Japan, 94.7% of carbapenem-resistant strains were of the IMP type, according to a survey conducted by the MHLW in 2011 [30]. In addition, carbapenem-resistant strains in 28 university hospitals in Japan from 2014 to 2016 were predominantly of the IMP type [31]. The majority of carbapenem-resistant strains in Japan are the IMP type or non-carbapenemase producing type of carbapenem-resistance, which has a lower minimum inhibitory concentration (MIC) for carbapenems than the KPC-2 and NDM types and often remains susceptible to aminoglycosides and fluoroquinolones as non-carbapenemase producing type of carbapenem-resistance [31, 32]. It is suggested that these are the reasons for the small difference in in-hospital mortality between CS and CR infections in the Japanese national survey and our study. The JANIS reported that the prevalence of IMP-type carbapenemases in carbapenem-resistant organisms varies among Western and other Asian countries. The remaining types of antibiotics susceptible to carbapenem-resistant infections may contribute to the reduction of mortality from carbapenem-resistant infections [33].

Another notable result of our study was the absence of carbapenem-resistant Acinetobacter species. In our study, the prevalence of carbapenem resistance was 0.9%. A total of 78 cases of carbapenem-resistant Enterobacteriaceae infections were reported, with the four main species *Enterobacter cloacae, Klebsiella aerogenes, Klebsiella pneumoniae,* and *Escherichia coli* accounting for 92.3% of cases. The top four species in the Enterobacteriaceae accounted for 83.7% of the carbapenem-resistant bacteria detected in our study, Pseudomonas aeruginosa was 9.3%, and no carbapenem-resistant Acinetobacter species. The incidence of carbapenem-resistant bacteria in Japan is less than 1%, with Enterobacteriaceae rather than Acinetobacter species being the primary threat [33–35]. The hospitals in Japan are required to report carbapenem-resistant bacterial infections to the MHLW, and 24 cases of carbapenem-resistant Acinetobacter species were reported during the year 2019, compared to the 2,333 cases of the carbapenem-resistant Enterobacteriaceae infections [34]. In Acinetobacter species, Multidrug-resistant Acinetobacter baumannii is particularly problematic worldwide, with reported hospital mortality rates ranging from 7.8 to 23% and ICU mortality rates from 10 to 43% [36]. In several randomized controlled trials comparing the efficacy of colistin with other antimicrobial combinations against Acinetobacter species, all-cause mortality exceeded 40% in both groups, with a maximum of 57.4% in several randomized controlled trials comparing colistin combination therapy with newer antimicrobial agents such as plazomicin, meropenem/vaborbactam, cefiderocol, etc. [37].

In this study, increased total costs, especially related to the medication, the laboratory test and the hospitalization, and prolonged LOS before culture test were observed in the carbapenem-resistant infections than the carbapenem-susceptible infections, but no difference in the LOS after culture test and in-hospital mortality. In general, prolonged patient hospitalization itself is also directly related to increased hospitalization costs. Our results suggest that reducing unnecessary hospital stays and using antimicrobial agents appropriately are rational ways to reduce the incidence of carbapenem-resistant organisms, control costs, and shorten hospital stays. If carbapenem-resistant genotypes of KPC-2 and NDM become widespread in Japan, the concern is not only an increased total cost of carbapenem-resistant infections but also increased LOS and in-hospital mortality.

## Limitations

This study has several limitations. First, it was difficult to accurately distinguish between patients with infections caused by carbapenem-resistant organisms and those with only colonization based on EMR information. Second, focusing on four major categories of diseases by the carbapenem-resistance bacteria. Third, the 55 hospitals affiliated with NHOs in Japan are covered, and the background circumstances may differ from the local settings in each of the various regions of the world. Since patients in Japan tend to have a longer LOS than those in many other countries, the situation may be different in other countries [38]. Fourth, there may have been unmeasured confounders. Future comparisons of the economic burden and clinical outcomes caused by carbapenem-resistant infections in multiple countries are warranted.

## Conclusions

This study revealed that the economic burden of CR infections was higher than CS infections. Detailed cost analysis showed significant differences in the categories of medication, laboratory tests, and hospital stay. It also showed that the several medical practices and cares before the culture test of using antibiotics, especially using carbapenem, ICU admission, and the others were associated with a longer total LOS and a higher total cost. To our knowledge, this is the first study to assess the economic burden of the CR infections using a large hospital database.

## .

## Supplementary Information

**Additional file 1: Table S1.** Baseline characteristics after adjustment with inverse probability of treatment weighting. **Table S2.** Death, LOS, cost of CR and CS infections after adjustment with inverse probability of treatment weighting. **Table S3.** Impact of carbapenem-resistant infections after adjustment with inverse probability of treatment weighting. **Figure S1.** Distribution of propensity scores before and after adjustment. **Appendix S1.** Sample size estimation for in-hospital mortality.

## Data Availability

The data that support the findings of this study are available from NHO Headquarters, but restrictions apply to the availability of these data, which were used under license for the current study, and so are not publicly available. Data are however available from the corresponding author NI, upon reasonable request and with permission of the NHO Headquarters.

## Abbreviations

AIC: Akaike information criterion
BMI: Body mass index
BT: Body temperature
CCI: Charlson Comorbidity Index
CRP: C-reactive protein
ICD-10: International Classification of Diseases, 10th Revision
ICU: Intensive care unit
MIA: Medical Information Analysis
NCDA: NHO Clinical Data Archives
NHO: National Hospital Organization
UTI: Urinary tract infection
VIM: Verona integron-encoded metallo-β-lactamase
WBC: White blood cell
