# Supplementary material for: Economic and clinical burden from carbapenem-resistant bacterial infections and factors contributing: a retrospective study using electronic medical records in Japan
Source: BMC Infect Dis. 2022 Jun 29;22:581. doi: 10.1186/s12879-022-07548-3 (PMC9241247; doi:10.1186/s12879-022-07548-3)
Supplement: Supplementary file 1 — Additional file 1: Table S1. Baseline characteristics after adjustment with inverse probability of treatment weighting. Table S2. Death, LOS, cost of CR and CS infections after adjustment with inverse probability of treatment weighting. Table S3. Impact of carbapenem-resistant infections after adjustment with inverse probability of treatment weighting. Figure S1. Distribution of propensity scores before and after adjustment. Appendix S1. Sample size estimation for in-hospital mortality. [file 12879_2022_7548_MOESM1_ESM.docx]

**Additional file 1**

**Economic and clinical burden from carbapenem-resistant bacterial infections and factors contributing: A retrospective study using electronic medical records in Japan**

[Table 1 Baseline characteristics after adjustment with inverse probability of treatment weighting. 2](#_Toc105869539)

[Table 2 Death, LOS, cost of CR and CS infections after adjustment with inverse probability of treatment weighting. 4](#_Toc105869540)

[Table 3 Impact of carbapenem-resistant infections after adjustment with inverse probability of treatment weighting 5](#_Toc105869541)

[Figure 1 Distribution of propensity scores before and after adjustment 6](#_Toc105869542)

[Appendix Sample size estimation for in-hospital mortality 7](#_Toc105869543)

# Table S1 Baseline characteristics after adjustment with inverse probability of treatment weighting

| **Variable** | **CS infections (N=3,493)** | **CR infections (N=86)** | **SMD** |
| --- | --- | --- | --- |
| Age (years), mean ±SD | 75.1 (13.1) | 75.1 (12.6) | <0.001 |
| Age (years), n (%) |  |  | <0.001 |
| 15–64 | 643 (18.4) | 16 (18.6) |  |
| 65–74 | 762 (21.8) | 19 (22.1) |  |
| ≥75 | 2088 (59.8) | 51 (59.3) |  |
| Sex, n (%) |  |  | <0.001 |
| Male | 2381 (68.2) | 59 (68.6) |  |
| Female | 1112 (31.8) | 27 (31.4) |  |
| BMI (kg/m^2^), mean ±SD | 20.8 (4.4) | 20.8 (4.5) | <0.001 |
| BMI (kg/m^2^), n (%) |  |  | 0.017 |
| <18.5 | 1869 (53.5) | 46 (53.5) |  |
| 18.5–25 | 1112 (31.8) | 27 (31.4) |  |
| ≥25 | 512 (14.7) | 13 (15.1) |  |
| WBC, mean ± SD | 11292.2 (6516.6) | 11292.2 (6460.2) | <0.001 |
| CRP, mean ± SD | 11.4 (8.5) | 11.4 (8.9) | <0.001 |
| BT, mean ± SD | 38.3 (0.9) | 38.3 (1.0) | <0.001 |
| Main diagnosis, n (%) |  |  |  |
| Pneumonia | 1873 (53.6) | 46 (53.5) | <0.001 |
| Sepsis | 576 (16.5) | 14 (16.3) | <0.001 |
| UTI | 953 (27.3) | 23 (26.7) | <0.001 |
| Biliary infection | 584 (16.7) | 14 (16.3) | <0.001 |
| Organism detected in culture test, n (%) |  |  |  |
| *Escherichia coli* | 742 (21.2) | 18 (20.9) | <0.001 |
| *Enterobacter cloacae* | 657 (18.8) | 16 (18.6) | <0.001 |
| *Klebsiella aerogenes* | 811 (23.2) | 20 (23.3) | <0.001 |
| *Klebsiella oxytoca* | 182 (5.2) | 4 (4.7) | <0.001 |
| *Klebsiella_pneumoniae* | 657 (18.8) | 16 (18.6) | <0.001 |
| *Proteus Mirabilis* | 93 (2.7) | 2 (2.3) | <0.001 |
| *Pseudomonas aeruginosa* | 350 (10.0) | 9 (10.5) | <0.001 |
| Reason for hospitalization, n (%) |  |  |  |
| Diseases of the circulatory system | 928 (26.6) | 23 (26.7) | <0.001 |
| Neoplasms | 612 (17.5) | 15 (17.4) | <0.001 |
| Diseases of the digestive system | 628 (18.0) | 15 (17.4) | <0.001 |
| Charlson Comorbidity Index, n (%) |  |  |  |
| 0 point | 2989 (85.6) | 74.0 (86.0) | <0.001 |
| ≧1 point | 504 (14.4) | 12 (14.0) | <0.001 |
| Comorbidity, n (%) |  |  |  |
| Myocardial infarction | 87 (2.5) | 2 (2.3) | <0.001 |
| Cerebrovascular disease | 1082 (31.0) | 27 (31.4) | <0.001 |
| Congestive heart failure | 676 (19.4) | 17 (19.8) | <0.001 |
| Rheumatic disease | 212 (6.1) | 5 (5.8) | <0.001 |
| Dementia | 303 (8.7) | 7 (8.1) | <0.001 |
| Diabetes without complications | 947 (27.1) | 23 (26.7) | <0.001 |
| Diabetes with complications | 230 (6.6) | 6 (7.0) | <0.001 |
| Mild liver disease | 209 (6.0) | 5 (5.8) | <0.001 |
| Moderate or severe liver disease | 83 (2.4) | 2 (2.3) | <0.001 |
| Peptic ulcer disease | 159 (4.6) | 4 (4.7) | <0.001 |
| Peripheral vascular disease | 138 (4.0) | 3 (3.5) | <0.001 |
| Chronic pulmonary disease | 223 (6.4) | 5 (5.8) | <0.001 |
| Paraplegia and hemiplegia | 44 (1.3) | 1 (1.2) | <0.001 |
| Renal disease | 186 (5.3) | 5 (5.8) | <0.001 |
| Cancer | 1149 (32.9) | 28 (32.6) | <0.001 |
| Metastatic carcinoma | 260 (7.4) | 6 (7.0) | <0.001 |
| HIV | 0 (0.0) | 0 (0.0) | – |
| Healthcare utilization |  |  |  |
| ICU admission | 782 (22.4) | 19 (22.1) | <0.001 |
| Surgical procedure | 1911 (54.7) | 47 (54.7) | <0.001 |
| Dialysis | 204 (5.8) | 5 (5.8) | <0.001 |
| Mechanical ventilation | 1216 (34.8) | 30 (34.9) | <0.001 |
| Immunosuppressive drug | 1230 (35.2) | 30 (34.9) | <0.001 |
| Antibiotics before the culture test, n (%) | 3047 (87.2) | 75 (87.2) | <0.001 |
| Carbapenem before culture test, n (%) | 995 (28.5) | 24 (27.9) | <0.001 |
| Number of beds, n (%) |  |  | <0.001 |
| <400 | 470 (13.5) | 12 (14.0) |  |
| 400–500 | 1271 (36.4) | 31 (36.0) |  |
| ≥500 | 1752 (50.2) | 43 (50.0) |  |
| Rout of admission, n (%) |  |  | <0.001 |
| From other wards | 0 (0.0) | 0 (0.0) |  |
| From home | 2778 (79.5) | 68 (79.1) |  |
| From other hospital | 542 (15.5) | 13 (15.1) |  |
| From long-term institutions | 126 (3.6) | 3 (3.5) |  |
| Others | 47 (1.3) | 1 (1.2) |  |

BMI, body mass index; CRP: c-reactive protein; CR, carbapenem-resistant; CS, carbapenem-susceptible; ICU, intensive care unit; PSM, propensity score matching; SMD, standardized mean difference; UTI, urinary tract infection.

# Table S2 Death, LOS, cost of CR and CS infections after adjustment with inverse probability of treatment weighting

| Variables | CS infections  (N=3,493) | CR infections  (N=86) | P-value |
| --- | --- | --- | --- |
| Death | 830 (23.8) | 22 (25.6) | 0.660 |
| Length of hospital stay (days), median (IQR) | | | |
| Total | 51 [31, 81] | 63 [39, 94] | 0.004 |
| Before culture test | 16 [7, 31] | 22 [10, 45] | 0.017 |
| After culture test | 29 [16, 50] | 30 [16, 66] | 0.253 |
| Cost (USD), median (IQR) | |  |  |
| Total | 22039 (14321–34108) | 26866 (16609–48751) | 0.004 |
| Inter-consultations | 154 (81–250) | 163 (95–309) | 0.193 |
| Medications | 2000 (889–4748) | 3371 (1580–8960) | <0.001 |
| Surgical procedures | 4042 (699–10079) | 3727 (482–12112) | 0.899 |
| Laboratory tests | 2189 (1350–3470) | 2491 (1666–4460) | 0.012 |
| Hospital stay | 11692 (7349–17267) | 13947 (9360–22470) | <0.001 |

CR, carbapenem-resistant; CS carbapenem-susceptible; LOS, length of stay; USD, U.S. dollar (1 USD=110 JPY, 2021).

# Table S3 Impact of carbapenem-resistant infections after adjustment with inverse probability of treatment weighting

|  | **Logistic regression**^†^ | |
| --- | --- | --- |
|  | **OR (95% CI)** | **P-value** |
| **Death** | 1.06 (0.50 to 2.24) | 0.880 |
|  | **Log-linear regression**^†‡^ | |
|  | **Percentage change (95% CI)** | **P-value** |
| **Total LOS** | 28.5% (23.6% to 33.4%) | <0.001 |
| **Total Cost** | 29.6% (24.6% to 34.6%) | <0.001 |

^†^ The impacts of carbapenem resistance infections were calculated against carbapenem susceptible infections as reference.

^‡^ Continuous outcomes of the LOS and cost were log-transformed. The estimated coefficient *β* of the carbapenem resistance was transformed as percent change per one-unit increase.

CI, confidence interval; CR, carbapenem-resistant; CS, carbapenem-susceptible; LOS, length of stay; ref, reference.


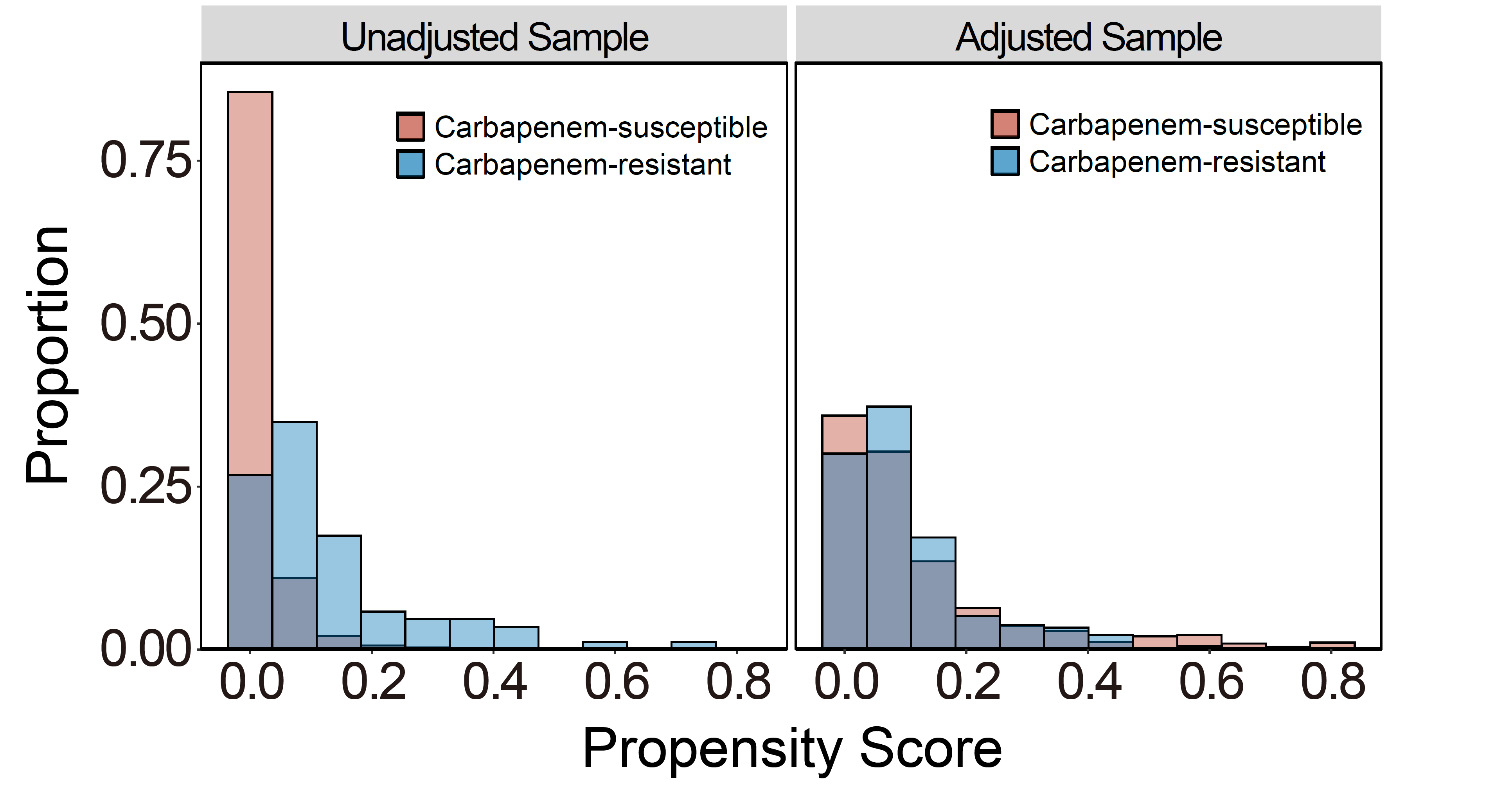


# Figure S1 Distribution of propensity scores before and after adjustment

Visualization and comparison of the propensity score distribution between carbapenem-susceptible infections and carbapenem-resistant infections before and after balancing and adjustment.

# Appendix S1: Sample size estimation for in-hospital mortality

The in-mortality between carbapenem-resistant (CR) infections and -susceptible (CS) infections described by Zhen et al. was used as a reference [2]. We estimated and grasped the sample size needed to show a significant difference in the in-hospital mortality in our study. Zhen et. al. described the in-hospital mortality between CR and CS infections by *Klebsiella pneumonia, Pseudomonas aeruginosa,* and *Acinetobacter baumannii.* In their study, *Klebsiella pneumonia* and *Acinetobacter baumannii* had a significant difference in the in-hospital mortality (*Klebsiella pneumonia*: CS 6.65%, CR 9.59%, absolute difference 2.94%, Pvalue = 0.024; *Acinetobacter baumannii*: CS 4.25%, CR 8.28%, absolute difference 4.03%, Pvalue = 0.003). *Pseudomonas aeruginosa* had the least absolute difference in the in-hospital mortality (CS 4.73%, CR 6.77%, absolute difference 2.03%) among these three species, and just the marginal significance (P = 0.052). If an equal sample size is anticipated in each group to detect more than a 2.03% reduction in the in-hospital mortality, the sample size limit required for 80% statistical power can be estimated bellow:

$$N_{1}=\{z_{1-\frac{\alpha}{2}}*\sqrt{\overline{p}*\overline{q}*(1+\frac{1}{k}})+z_{1-\beta}*\sqrt{p_{1}*q_{1}+(\frac{p_{2}*q_{2}}{k}}){\}}^{2}/\Delta^{2}$$

$q_{1}=1-p_{1}$, $q_{2}=1-p_{2}$

$\overline{p}=\frac{p_{1}+kp_{2}}{1+K}$, $\overline{q}=1-\overline{p}$

$$\Delta=\left| p2-p1 \right|$$

then

$$N_{1}=\{1.96*\sqrt{0.0575*0.9425*(1+\frac{1}{1}})+0.84*\sqrt{0.0473*0.9527+(\frac{0.0677*0.9323}{1}}){\}}^{2}/{0.0204}^{2}$$

$\boldsymbol{N}_{\boldsymbol{1}}\boldsymbol{≒2041}$**,** $\boldsymbol{N}_{\boldsymbol{2}}\boldsymbol{=k*}\boldsymbol{N}_{\boldsymbol{1}}\boldsymbol{≒2041}$

*p*_1_, *p*_2_: proportion (incidence) of groups #1 and #2

*N*_1_, *N*_2_ : sample size for group #1 and #2

*α* : probability of type I error (usually 0.05), *β* : probability of type II error (usually 0.2)

*z* : critical *Z* value for a given *α* or *β*

*k* : ratio of sample size for group #2 to group #1

**Reference**

1. Rosner, B. (2010) Fundamentals of Biostatistics. 7th Edition, Brooks Cole, Salt Lake City, Utah, 303.
2. Zhen X, Stålsby Lundborg C, Sun X, Gu S, Dong H. Clinical and Economic Burden of Carbapenem-Resistant Infection or Colonization Caused by Klebsiella pneumoniae, Pseudomonas aeruginosa, Acinetobacter baumannii: A Multicenter Study in China. Antibiotics. 2020;9:514. doi:10.3390/antibiotics9080514.
